# Malmö Breast ImaginG database: objectives and development

**DOI:** 10.1117/1.JMI.10.6.061402

**Published:** 2023-02-08

**Authors:** Victor Dahlblom, Magnus Dustler, Anetta Bolejko, Predrag R. Bakic, Henrik Granberg, Kristin Johnson, Daniel Förnvik, Kristina Lång, Anders Tingberg, Sophia Zackrisson

**Affiliations:** a Lund University, Department of Translational Medicine, Diagnostic Radiology, Malmö, Sweden; b Skåne University Hospital, Department of Medical Imaging and Physiology, Malmö, Sweden; c Lund University, Department of Translational Medicine, Medical Radiation Physics, Malmö, Sweden; d Skåne University Hospital, Radiation Physics, Malmö, Sweden; e Skåne University Hospital, Unilabs Breast Centre, Malmö, Sweden

**Keywords:** artificial intelligence, big data, breast cancer screening, database management systems, mammography

## Abstract

**Purpose:**

We describe the design and implementation of the Malmö Breast ImaginG (M-BIG) database, which will support research projects investigating various aspects of current and future breast cancer screening programs. Specifically, M-BIG will provide clinical data to:

1. investigate the effect of breast cancer screening on breast cancer prognosis and mortality;
2. develop and validate the use of artificial intelligence and machine learning in breast image interpretation; and
3. develop and validate image-based radiological breast cancer risk profiles.

**Approach:**

The M-BIG database is intended to include a wide range of digital mammography (DM) and digital breast tomosynthesis (DBT) examinations performed on women at the Mammography Clinic in Malmö, Sweden, from the introduction of DM in 2004 through 2020. Subjects may be included multiple times and for diverse reasons. The image data are linked to extensive clinical, diagnostic, and demographic data from several registries.

**Results:**

To date, the database contains a total of 451,054 examinations from 104,791 women. During the inclusion period, 95,258 unique women were screened. A total of 19,968 examinations were performed using DBT, whereas the rest used DM.

**Conclusions:**

We describe the design and implementation of the M-BIG database as a representative and accessible medical image database linked to various types of medical data. Work is ongoing to add features and curate the existing data.

## 1 Introduction

Large, representative databases are fundamental in the field of medical imaging for both research and quality assurance. Such databases enable continuous evaluation of diagnostic performance and provide raw data for risk stratification models of diverse conditions. Additionally, the emerging use of artificial intelligence (AI) for automated or improved image interpretation requires large quantities of medical images paired with accurate diagnostic information to train and validate AI algorithms.

This paper describes the development and setup of the Malmö Breast ImaginG (M-BIG) database, which is focused on radiological imaging in the context of breast cancer screening. M-BIG will aid the development and validation of new AI-based image analysis algorithms and provide data for investigating radiological risk models and breast cancer screening outcomes. The database is designed as a complete, consecutive collection of screening and diagnostic digital mammography (DM) and digital breast tomosynthesis (DBT) images paired with curated demographic and diagnostic information.

### 1.1 Breast Imaging in Screening

Breast cancer screening has generally been organized in the same manner since its introduction, offering a generic method and interval to all women and reading all examinations with the same protocol. Some screening programs employ an age-based stratification of the screening interval, but screening is usually not individualized to personal risk factors. The technology itself has continually improved, the most prominently the transition from screen film to DM. Evidence suggests that DM screening has limited sensitivity and specificity, missing 10% to 25% of breast cancers and recalling 6 to 20 healthy women for each diagnosed cancer.^1^^,^^2^ Double reading, in which two breast radiologists independently read each examination, is practiced in Sweden and many other European countries to increase both sensitivity and specificity, but it increases the overall workload.^3^ DBT—a pseudo-3D development of DM—has been proposed and evaluated as a screening method to replace or complement DM and has been introduced in some screening programs. Evidence suggests that DBT provides greater accuracy than DM.^4^

The true effect of mammography screening has been repeatedly debated. Breast cancer detection through mammography screening is believed to reduce breast cancer mortality;^5^ still, this assumption is based on randomized controlled trials conducted several decades ago. Since then, new breast cancer treatments and an improved mammographic technique have emerged, which adds to the complexity of evaluating the true effect of screening in today’s environment. Nevertheless, it is important to understand the effect of screening on mortality, to identify potential weak links in the clinical care chain, and to better understand why women die from their breast cancer, not least for the purpose of designing future—potentially individualized—screening strategies.

### 1.2 Computer-Assisted Detection and Artificial Intelligence

Since the 1990s, computer-aided detection (CAD) systems for screening review have been available that aim to increase performance and reduce reading time, but they have not been widely adopted due to their reduced specificity.^6^ The emergence of deep-learning and convolutional neural networks has inspired hope that AI-based CAD will perform better.^7^ AI has potential to greatly affect the breast imaging workflow if used in conjunction with a human reader as a decision aid or as a stand-alone tool for tasks such as risk prediction. Furthermore, many mammography clinics do not have enough breast radiologists, so adding AI to reduce their work burden is attractive and perhaps crucial to sustain screening programs. Reading time is even more critical when DBT is used, as reading times will be prolonged (approximately doubled compared to DM).^8^

The evolution of deep-learning-based AI algorithms to identify cancer in medical imaging highlights the importance of access to large collections of high-quality data, as a large amount of data is crucial for successful algorithm training. Breast cancer screening by mammography has gained special attention due to both increasing demand (as described above) and the availability of training data, including both normal and abnormal cases.

In addition to training AI systems, databases are very important for the performance evaluation. Without large, representative databases of annotated medical images (including access to high-quality diagnostic information), it is impossible to assess the performance of AI (or any other image analysis tool) in breast cancer screening programs, due to the relatively low prevalence of the disease.

### 1.3 Risk Stratification and Individualized Screening

Another important development is individualized breast cancer screening.^9^ Polygenic risk scores that combine hereditary and lifestyle factors with breast density could optimize the imaging method and screening interval for specific risk groups.^10^ Such models could be refined using radiological risk profiles based on the analysis of parenchymal texture and other image findings.^11^^,^^12^ Large sets of images are crucial in evaluating which image-based tissue features that are of clinical importance. For example, a high percentage of fibroglandular (i.e., dense) tissue is an independent risk factor for breast cancer,^13^ and the amount of dense tissue can be automatically measured from the image. Furthermore, repeated imaging contains valuable prognostic information on the growth rate of breast tumors, which is currently rarely used in treatment planning. In the right circumstances, it is possible to track tumor progression and calculate the tumor growth rate based on findings from previous images.^14^ Risk prediction based on a potential change in breast density and parenchymal texture over a longer period (i.e., repeated screening rounds) has not yet been extensively investigated with AI.

### 1.4 Breast Imaging Databases

Conducting research is impractical on data directly from a clinical radiological information system (RIS) or picture archiving and communication system (PACS), as accessing large volumes of image data can be slow and potentially compromise the system performance of normal clinical routines. Furthermore, a large part of the data is distributed in many different systems and cannot easily be accessed or linked. Thus it is necessary to create separate research databases that enable quick, efficient access to data.

A number of databases of screening mammography examinations are already available, including the British database OMI-DB,^15^ which was collected via a safe pipeline and includes more than 3 million images and associated data from about 170,000 women aged 50 to 70 years who were screened at three institutions in 2010 to 2020. All screening-detected and interval cancers were collected, and normal cases were restricted to a random subset. Cancer locations are marked with rectangles on the images.

Another database is the Swedish Cohort of Screen-Aged Women (CSAW), which includes images and breast cancer diagnoses of all women in the screening age range (40 to 74 years) of the Stockholm region’s screening program in 2008 and 2015.^16^ This includes almost 1.2 million examinations (all acquired with Hologic equipment) of ∼500,000 women. Some of the cancers were annotated by outlining them in the images.

No single database is enough, however. Using data from different sources makes both the training and evaluation of AI models more robust, as various populations as well as images with diverse technical properties are included, minimizing the risk of bias.

### 1.5 Objectives

The overall objective of this project was to build the M-BIG, a large database containing a wide range of DM and DBT examinations from a single population more than 15 years along with extensive, curated demographic, and clinical data. M-BIG will be used in research projects investigating various aspects of current and future breast cancer screening programs. Its specific objective is to provide data for:

1. investigating the effect of breast cancer screening on breast cancer prognosis and mortality;
2. developing and validating AI and machine learning in breast image interpretation; and
3. developing and validating image-based radiological breast cancer risk profiles.

Beyond these aims, the database addresses a number of additional needs; for example, it will provide an accessible source of images for human reader studies.

## 2 Methods

The M-BIG database contains DM and DBT examinations of women at the mammography clinic in Malmö from the introduction of DM in 2004 through 2020. This includes screening examinations, examinations of women recalled from screening, and examinations of women referred due to symptoms. For women diagnosed with breast cancer, it includes all examinations performed before and after surgery as well as during and after treatment. Subjects may be included multiple times and for diverse reasons.

### 2.1 Development Strategy

The development strategy of the M-BIG database relied on team-based planning and shared decision-making. The project was initially handled by a small work group that investigated various options for the database’s scope and structure. The more active development process began after the establishment of a steering group that comprised researchers in various diagnostic imaging professions, experienced IT support, and a dedicated project manager. The steering group was responsible for identifying the database’s desired components, devising a potential interface to other clinical databases, and discussing potential user features and functionalities.

**Fig. 1:**
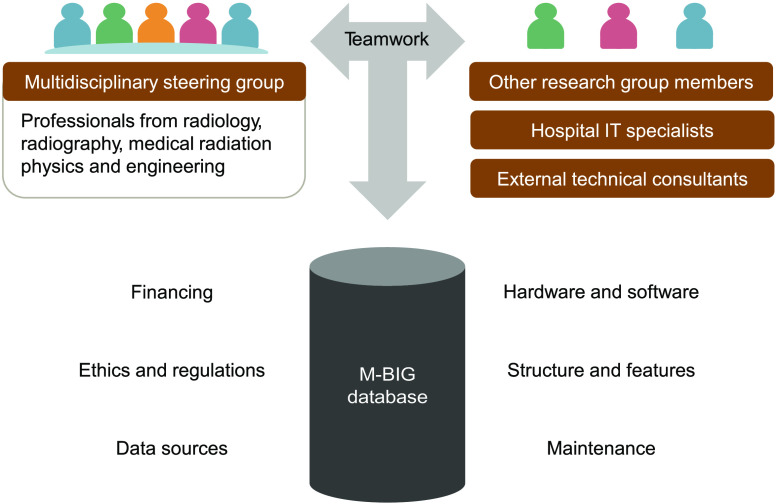
The development process based on inclusive project management with shared decision-making.

Figure 1 illustrates the development process. The initial step identified three core components, including the computational infrastructure, financing, and ethical and regulatory approval. A project manager oversaw the project’s progress, documentation, and follow-up. Planning, task distribution, and the mapping of each step of the development process were accomplished via teamwork and shared decision-making, reflecting our institution’s successful, inclusive approach to management.

**Fig. 2:**
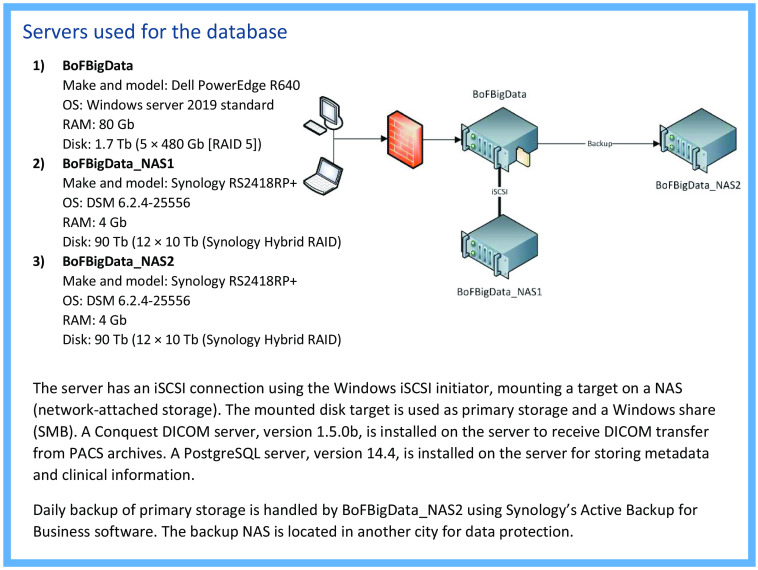
Hardware and software of the database’s servers with a schematic illustration of its structure and connections. RAID, redundant array of independent disks.

In accordance with the Swedish Ethics Review Act (2003:460), the project was approved by the ethics review authority (Dnr 2018/322, 2021-00769). The European General Data Protection Regulation (EU 2016/679) was also taken into account and influenced, e.g., data security, access to the data, and the conditions for consent. (More details on ethics and data protection are provided in Sec. 2.6.) A technical infrastructure was established for image transfer, storage, and back-up as well as for matching with relevant clinical records and register data. The hosting of the database was arranged within the hospital, providing compatible IT support and a data protection policy. The servers that support the database are described in Fig. 2.

### 2.2 Population and Data Sample

In 2004, breast cancer screening examinations in Malmö changed from screen-film mammography to DM. Due to changes in the national recommendations, the age groups screened varied before 2010. From 2004, women aged 50 to 74 years were invited biannually, but the lower age limit was reduced to 48 years in 2007 and 40 years in 2009. Since 2010, all women aged 40 to 74 are invited every 18 months until age 55 and every 24 months thereafter.

The majority of the included screening examinations are therefore of women aged 40 to 74, but a smaller number of younger and older women referred for symptoms are also included. The average participation rate in breast cancer screening in Sweden is 81%.^17^ The uptake area for the database population includes the Malmö metropolitan area and outlying communities that had been screened centrally in the Mammography Clinic at Skåne University Hospital (SUS) in Malmö. SUS Malmö has also been a referral center for nearby screening centers, with some changes over the time span. (The mammography Clinic at SUS Malmö was formerly part of the hospital’s Radiology Department, but it has been privately run by Unilabs AB under a contract with Region Skåne since April 1, 2009.)

### 2.3 Data Sources

The database focuses on breast cancer screening, but other mammographic examinations were included, as they include important information about cancers diagnosed or missed by screening. Data on cancers in women not taking part in screening are also important, for example, for evaluating how the screening program affects the entire target population. Furthermore, preoperative tumor localizations and specimen examinations provide important information for identifying the location of tumors. Ultrasound examinations are not currently included, as that modality is very user dependent, and examinations are often hard to interpret retrospectively. Magnetic resonance imaging (MRI) examinations are not currently included in the database.

A breast cancer screening examination usually includes two DM views of each breast [craniocaudal and mediolateral-oblique (MLO)]. The examinations are read by two specialized breast radiologists, and difficult or discrepant cases are flagged for consensus discussion. Each radiologist codes each examination with a score of 1 to 5 [(1) normal, (2) benign findings, (3) nonspecific findings where malignancy cannot be excluded, (4) findings suspicious for malignancy, and (5) malignant findings].^18^ The scoring largely resembles the British and Australian classification systems.^19^^,^^20^ Compared to BI-RADS, there is a difference in score 3, which has a higher cancer suspicion level than in BI-RADS and usually is biopsied.^21^ Further, the implementation of the scoring differs, e.g., a score is set already based on the screening examination before recall and a new separate score is set at the recall examination.

Additional DM views, e.g., mediolateral and spot views, (and usually ultrasound) are often added at recalls and diagnostic examinations. If needed and available, one or more DBT views may be added. Suspicious lesions are biopsied with either fine needle (cytology), core needle, or vacuum biopsy (pathology). The examination results, including pathology or cytology results, are reported in free-text comments to the referring physician and in the medical records. Cancer diagnoses are compulsory reported to the Cancer Registry.^22^

All images are stored as Digital Imaging and Communications in Medicine (DICOM) images, including full DICOM headers, in a clinical PACS (Sectra AB, Linköping, Sweden), and all screen reading results and radiological reports are kept in a RIS (Sectra).

Inclusion was handled practically by querying the PACS/RIS about all examinations with relevant code sequences and/or relevant code descriptions performed from January 1, 2004 through December 31, 2020. Code sequences and code descriptions were initially selected from the internal set of PACS/RIS codes if they contained the words mammography, tomosynthesis, or breast, and irrelevant ones were manually removed. The code sequences considered to be relevant to the study are shown in Table 1. Some codes may overlap due to changes in the internal code system over time.

**Table 1:** Examination codes included in the database.

Code	Description
66000	Mammography complete examination (diagnostic mammography)
66001	Review, report, and/or demo of external mammography
66002	Review, report, and/or demo of internal mammography
66030	Tomosynthesis
66059	Mammography, recalled from screening
66061	Diagnostic breast tomosynthesis
66200	Screening mammography
66230	Screening breast tomosynthesis
66300	Galactography
66500	Stereotactic breast biopsy, fine needle
66507	Stereotactic breast biopsy, screening
66550	Stereotactic breast biopsy
66551	Mammographic breast biopsy, core
66560	Mammographic breast biopsy
66600	Preoperative tumor localization mammography
66607	Preoperative tumor localization directed by x-ray, screening
66700	X-ray assisted breast biopsy, nonstereotactic
66800	Breast specimen x-ray, preoperative
66802	Mammographic examination of biopsy
66807	Breast specimen x-ray, postoperative

Sweden has a well-developed system of personal identification numbers, which makes it easy to link information from various sources to the same person. Information on all breast cancer diagnoses is registered in the Swedish national cancer registry, including diagnosis, date, and some basic characteristics of the cancer.^22^ For more convenient access, the regional cancer registry is being used initially, including all cancers diagnosed in the region. Additional information, specifically regarding tumor stage, time until treatment, treatment provided, and results, is available from Sweden’s National Quality Register Breast Cancer (NKBC) registry. More detailed information on cancer characteristics, including tumor type and receptor status, is available in the pathology reports in each patient’s medical record.

**Fig. 3:**
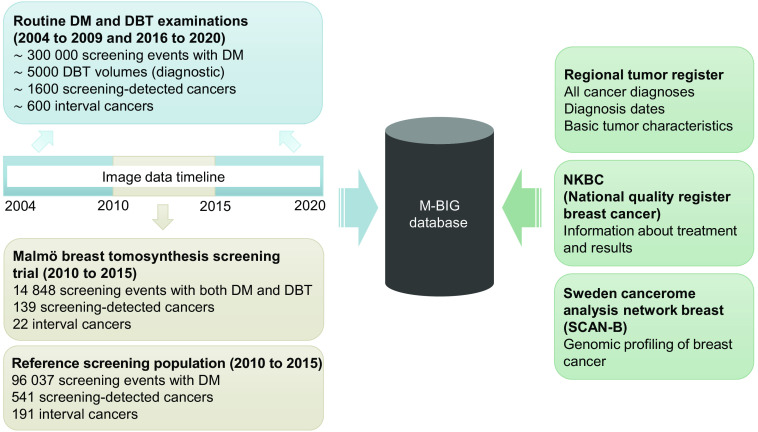
Sources of images and other data for the M-BIG database. DM, digital mammography and DBT, digital breast tomosynthesis.

A number of scientific studies have included various subgroups of the population, and additional information is potentially available for those subgroups as shown in Fig. 3. The Malmö Breast Tomosynthesis Screening Trial (MBTST), conducted in 2010 to 2015,^23^ included 14,848 women who were examined with single-view (MLO) wide-angle DBT in addition to conventional two-view DM; they will be included in the database. Valuable clinical information on this population as well as a reference population was collected in a structured form during two follow-up screening rounds (unpublished data).^24^

A local biobank, the Sweden Cancerome Analysis Network-Breast (SCAN-B), includes genomic analyses of all newly diagnosed cancers in consenting women in the region since 2010 (except for the smallest tumors, when the amount of spare tissue is inadequate for biobank use after clinical routine analyses).^25^ Similar data are also available for 2007 to 2010 from a previous project, all breast cancer in Malmö (ABiM).^26^

### 2.4 Database Structure

Because the database will continually be expanded with more data (including both relevant clinical data from different registries and, eventually, data from an expanded inclusion period), it is necessary to maintain a key that identifies each person. However, this key should be accessible and used only when absolutely necessary; therefore, it must be saved separately from the rest of the data. Consequently, we constructed the database of metadata and clinical information in three separate layers as described below; the images are stored as separate, unmodified files.

1. *Main database*. Contains most of the information. Used for browsing the data, gathering information for research, and selecting cases for subprojects. All identifiers are pseudonymized, i.e., identifiers are changed to randomly generated identifiers, but a key is saved to allow future linking to the original identifiers. Birth dates are randomly time shifted up to ±100 days, but examination dates are left unchanged. Contains no file paths but rather a path ID unique to each file.
2. *Path database*. Contains the path ID and file path. Used to locate the DICOM file when it has been selected for export.
3. *Key database*. Contains keys to link pseudonymized IDs and actual IDs. Includes both personal IDs and DICOM IDs. Additionally, a nonpseudonymized copy of some of the basic data is kept here.

All the relevant information was gathered into database tables using the open-source database system PostgreSQL (PostgreSQL Global Development Group). Structured Query Language (SQL) was selected because it is relatively easy to implement and allows complex queries for data search and aggregation. As SQL is a very popular database language, numerous tools are available to facilitate the construction of queries; MATLAB Database Explorer (MathWorks Inc., Natick, Massachusetts) is currently available in our environment. The ability to use custom-written SQL queries can be very valuable for more advanced tasks.

When extracting subpopulations for specific projects or for data sharing with collaborating parties, the data can be repseudonymized with a project-specific key. For technical reasons, a key must be created for use during the pseudonymization process. The key can be kept for the project if necessary (and ethically approved); otherwise, it can be deleted before transferring the data, and thus the data will be anonymous. Optionally, the repseudonymization can remove all dates, or if the timespan between examinations is important, all dates for each woman can be time shifted with a random person-specific offset, e.g., up to ±100 days, i.e., the interval between examinations will be unchanged.

### 2.5 Collection, Organization, and Curation of Data

**Fig. 4:**
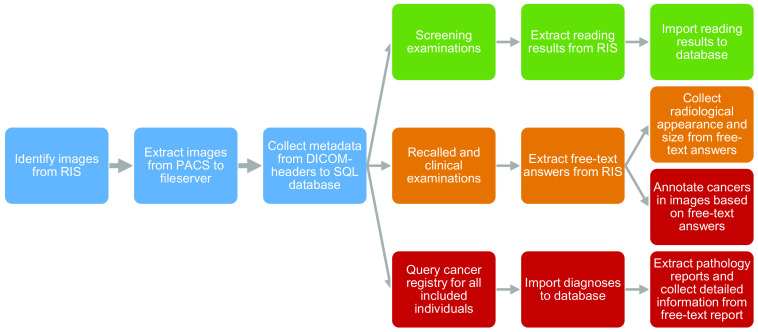
Flowchart of the steps in collecting and curating data for the database. Green: all cases; orange: recalled from screening and diagnostic images; red: cancer cases. RIS, radiology information system; PACS, picture archiving and communication system; DICOM, digital imaging and communications in medicine; and SQL, Structured Query Language.

Figure 4 illustrates the process of collecting images and clinical information as well as curating them for efficient use in research. The transfer of images from PACS was performed with technical help from Sectra. Although all breast imaging examinations from recent years are stored in a specific mammography PACS, some of the oldest images were stored only in the common radiology PACS. Organizational changes caused some differences in the coding of images during the period as shown in Table 1.

DICOM images were extracted from the PACS archive. For technical reasons, they were stored as unmodified DICOM files (including personal data) on a file server within the hospital network and were thus protected by the hospital’s firewall. Storage as separate DICOM files is relatively easy to set up and ensures that the images can easily be accessed and exported through standard network file sharing.

To enable efficient database queries, a registry was created of all stored DICOM files using custom-developed MATLAB scripts that scanned the files, read the DICOM metadata, and saved information about each file in a PostgreSQL database. Basic personal data (e.g., personal identification number, age at examination, and study date) were saved with identifiers of the DICOM file, including universally unique identifiers (UIDs) of instance, series and study, and path to the DICOM file. Other data were also saved, in particular exposure information, so that the included criteria can be used to refine searches as well as to facilitate efficiently accessing and aggregating such information for research purposes. Additionally, a separate MATLAB structure variable containing all the metadata was saved to the database to make it possible to add additional fields without opening individual files.

Clinical information from diverse sources is included in the PostgreSQL database as separate database tables. For screening examinations, the reading results are extracted from the RIS’s mammography screening module and imported to the database. Specific reader information is saved for both readers regarding scores, flags for discussion, and recalls. The results of any consensus discussions and final decisions are also included.

Information on breast cancer diagnoses, including a number of basic variables, was imported from the cancer registry for all women with extracted images. For recalled and diagnostic cases, the original answers are saved as free text; thus they are complicated and time-consuming to access in a structured fashion. The free-text comments have been included in the database, and this information can be used, for example, to annotate the location of cancers. Additional cancer characteristic data were available for the majority of screening-detected and interval cancer cases from 2010 to 2015; the data were extracted from free-text pathology reports as part of the MBTST and related projects.

### 2.6 Ethics, Regulations, and Data Security

In Sweden, all research studies involving human subjects must be approved by the Swedish Ethical Review Authority. Additionally, Region Skåne as the data owner requires a separate approval for data access, including many of the same elements as in the ethics review board application.

As this project includes only retrospective data, the risk to the included women is limited to privacy concerns. The application described the planned use of personal data, expected value of the research, potential risks, and means to minimize those risks. The privacy concerns can be addressed by anonymizing the data, but, because the database will be expanded with more data in the future, it is necessary to maintain a key. Consequently, an approach using pseudonymized data was selected to minimize privacy concerns. Furthermore, the data are protected by the same IT security that safeguards the clinical data. The potential benefit is mainly at the population level on the assumption that the project can improve breast cancer screening, diagnosis, or treatment. As the risk to participants is very small, the ethics review board waived the need for informed consent. An announcement describing the ability to opt out is available in Ref. 27.

During the first phase of the project, the legal structure that regulates the handling of personal information was changed when the common European General Data Protection Regulation (GDPR) was implemented, superseding the previous national laws. The introduction of the GDPR inspired a deeper awareness of privacy concerns and the development of additional structures to address them. This required added administrative work but probably resulted in a more secure database with even greater consideration of personal privacy.

Because the images are stored unmodified, the pseudonymization must be performed during export from the database; all identifying information is then replaced by pseudonymized information. The pseudonymization is performed with a MATLAB script and includes only the image data and a limited selection of vital DICOM metadata to maintain personal privacy at the highest possible level. All DICOM UIDs are pseudonymized. When shared with other researchers, the data can be exported in a pseudonymized state using a project-specific key. The key can be removed if no longer needed or in the case of a security breach; thus, the data will become anonymous.

## 3 Results

### 3.1 Characteristics of the Population

To date, the database contains 451,054 examinations from 104,791 women; 95,258 unique women were screened during the inclusion period. A total of 19,968 examinations were performed using DBT, the rest using DM. Table 2 shows the number of included examinations for each included examination code.

**Table 2:** Number of examinations for each code identified for inclusion in the database.

Code	Description	*Number*
66000	Diagnostic mammography	66,408
66001	Review, report, and/or demo of external mammography	284
66059	Mammography, recalled from screening	9813
66061	Diagnostic breast tomosynthesis	4190
66200	Screening mammography	343,100
66230	Screening breast tomosynthesis	15,778
66300	Galactography	430
66500	Stereotactic breast biopsy, fine needle	256
66507	Stereotactic breast biopsy, screening	498
66550	Stereotactic breast biopsy	181
66551	Mammographic breast biopsy (core)	1,387
66600	Preoperative tumor localization mammography	783
66607	Preoperative tumor localization directed by x-ray, screening	175
66800	Breast specimen x-ray, preoperative	5,264
66802	Mammographic examination of biopsy	126
66807	Breast specimen x-ray, postoperative	829
	Other (codes with < 100 items)	190
	Missing code sequence	1,362

**Fig. 5:**
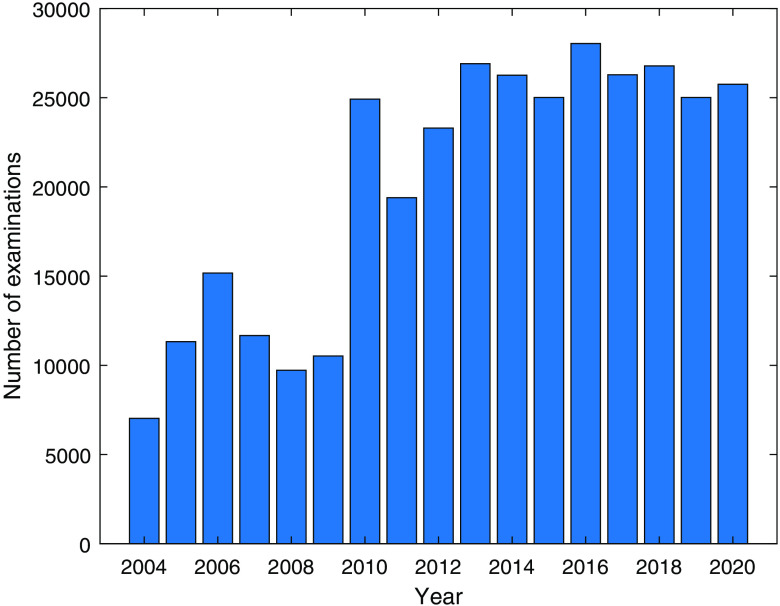
Number of screening examinations in the M-BIG database by year.

**Fig. 6:**
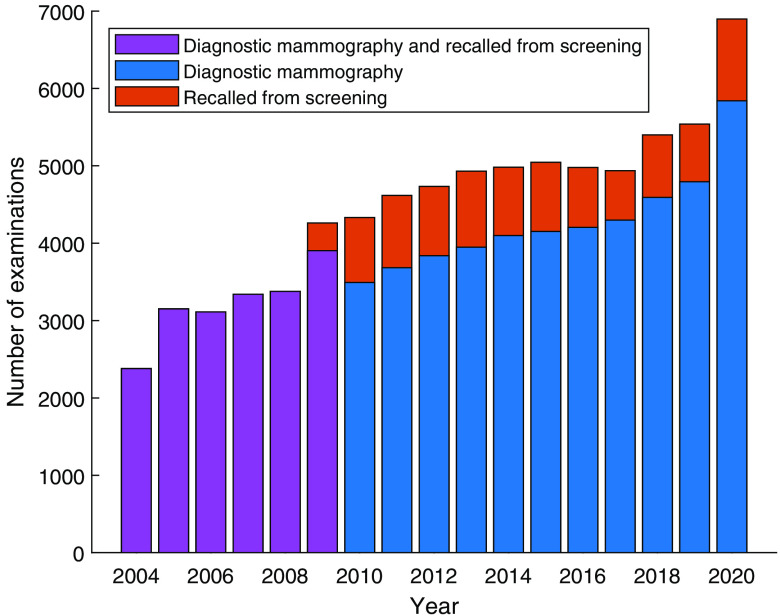
Number of diagnostic mammography examinations and mammography examinations of women recalled from screening per year. Separate data on those recalled from screening were not available before 2009, as a separate examination code was not used then.

**Fig. 7:**
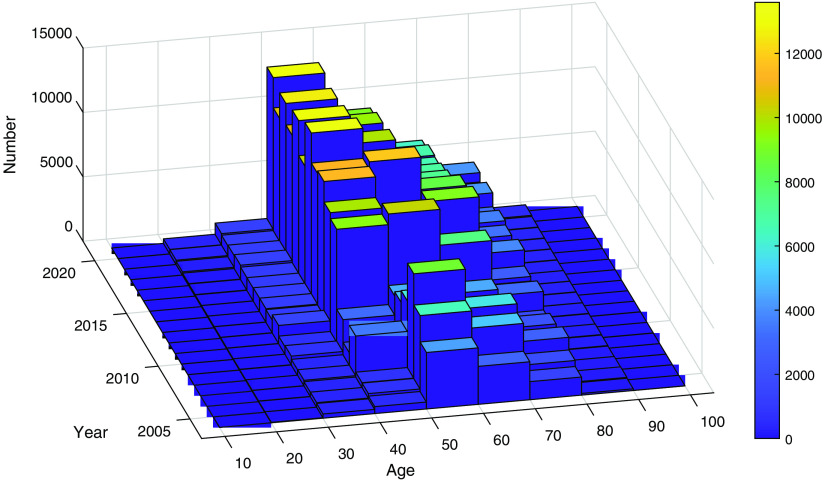
Total number of examinations by age and year (including screening examinations, diagnostic examinations, and recalls from screening).

The number of examinations per year (Fig. 5) increased significantly in 2010, when the screening program was expanded to include women aged 40 to 49. Figure 6 shows the yearly number of diagnostic examinations and examinations of women recalled from screening. Figure 7 shows the distribution of all included examinations by year and age, revealing a distinct peak representing women aged 40 to 49 after 2010. As their screening interval is shorter for older groups, a larger number of examinations per year is expected in this group than in others.

**Fig. 8:**
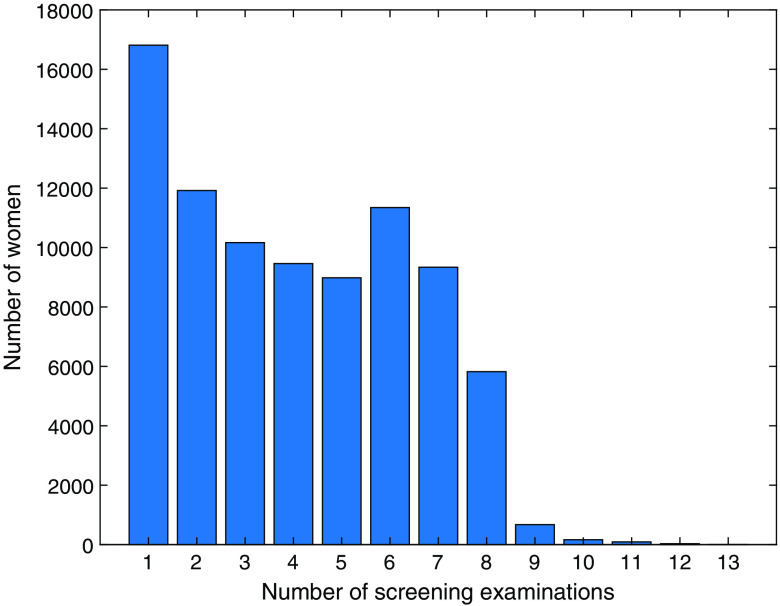
Number of screening examinations per woman.

**Fig. 9:**
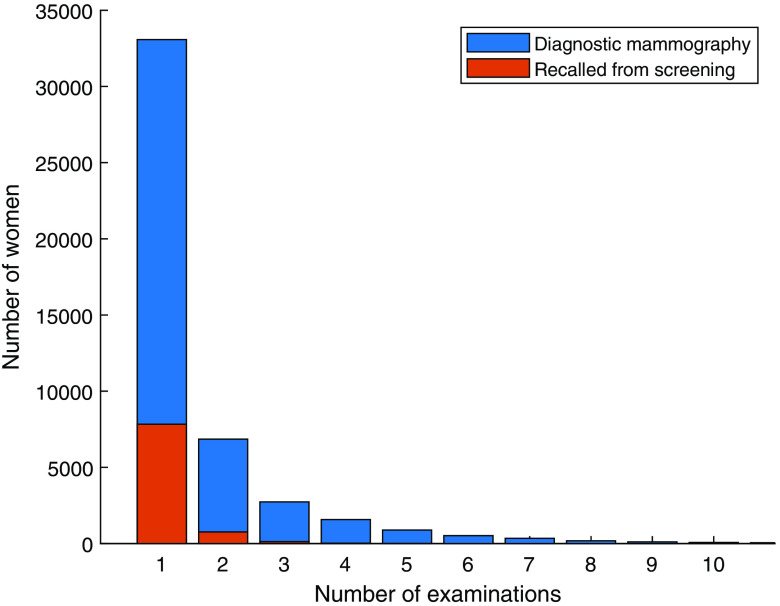
Number of diagnostic mammography examination per woman by circumstance (diagnostic mammography or recall from screening). Note that mammography examinations after recall from screening before 2009 cannot be separated, as a separate examination code was not used then.

Figure 8 shows the number of screening examinations per woman. A plurality of the women had undergone only one examination, which can partly be explained by the fact that some women were screened for their first time during the last 18 months of the inclusion period. Women who chose to drop out of screening after the first examination may also be in this group. As the database spans 17 years, a woman attending screening at normal intervals (18 or 24 months depending on age) would have a maximum of nine examinations. In selected cases, however, a shorter interval may be used, so some women have more examinations. The number of diagnostic mammography examinations and mammography examinations after recall from screening per woman is presented in Fig. 9.

**Fig. 10:**
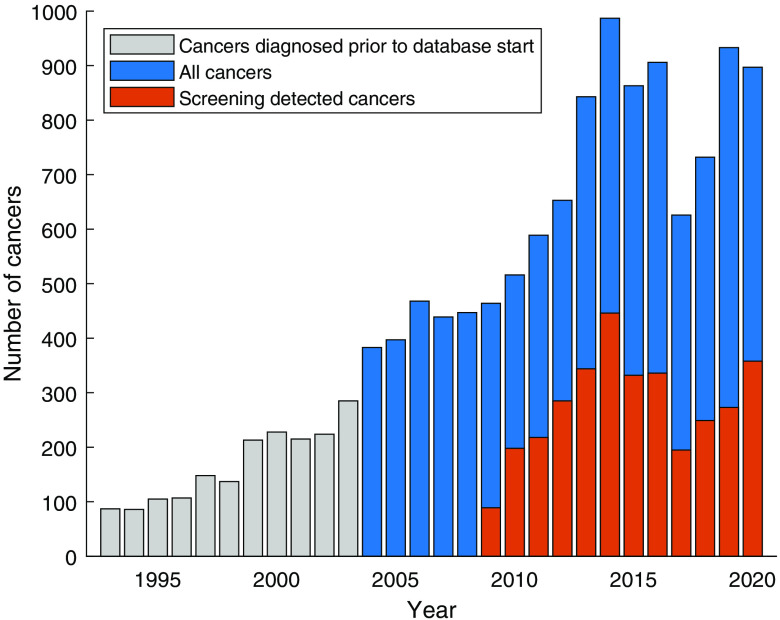
Number of breast cancers diagnosed per year among women in the database. Note that screening-detected cancers before 2009 cannot be separated, as a separate examination code was not used then.

Figure 10 shows the number of breast cancer diagnoses per year among all women represented in the database. This also includes diagnoses prior to the time span included in the database (going back to 1993). The population is defined as women with any examination in the database beginning in 2004; thus, in the figure, the population is smaller and younger in the first years, and the progressive increase is expected.

**Fig. 11:**
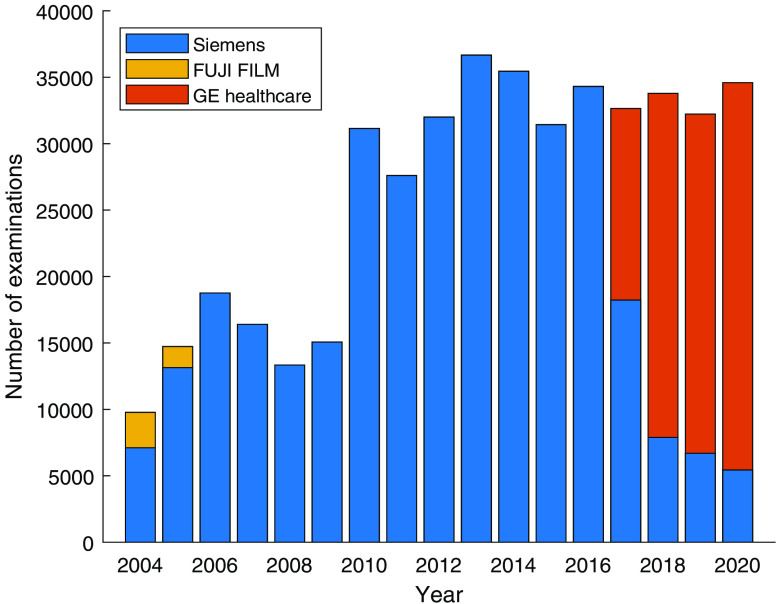
Number of examinations performed per year by equipment manufacturer.

The database includes examinations performed with equipment from various manufacturers as shown in Fig. 11. Of the DBT examinations, the vast majority was performed on Siemens equipment (Mammomat Inspiration). In Table 3, the proportion of women invited to breast cancer screening who attended is shown for each year.

**Table 3:** Breast cancer screening attendance rate per year.

Year	Attendance rate (%)
2004	81
2005	84
2006	84
2007	83
2008	86
2009	83
2010	78
2011	72
2012	75
2013	79
2014	76
2015	81
2016	78
2017	78
2018	78
2019	78
2020	63

## 4 Discussion

We have built a research database containing all DM and DBT examinations (both screening and diagnostic) of women examined in Malmö more than 17 years. The three main aims of the M-BIG are to provide suitable high-quality data for investigating the effect of screening on breast cancer prognosis and mortality, to develop and validate breast imaging AI, and to develop and validate image-based radiological risk profiles.

This database contains examinations performed over many years using equipment from various vendors and includes all mammography examinations performed at a single institution, regardless of age, screening attendance, or cancer status. It also includes DBT examinations from the DBT screening study MBTST as well as all diagnostic DBT examinations performed on recalled or referred women.

Building the database took longer than initially expected, and many steps were more complicated than foreseen, e.g., identifying relevant key persons in the organization and establishing cooperation with them. The project’s advancement depended crucially on the formation of a multidisciplinary steering group including IT staff from the hospital and on close cooperation with the PACS manufacturer, Sectra.

This is an early report on the database, and development and expansion of the database is ongoing. Currently, the database proper contains only images for the included women. Cancer data from the regional cancer registry and the reading data are stored as separate files but are yet to be imported into the PostgreSQL database. Pathology information for parts of the population (in particular for women taking part in the MBTST and for the years 2010 to 2015) is also available as separate files but has not been imported into the PostgreSQL database. M-BIG is designed to be easily expanded with additional data and to provide readily linkable image data, clinical data, and pathological data.

By numbers, the database is dominated by screening examinations, but it also contains a substantial proportion of diagnostic examinations and recalls from screening (Figs. 5 and 6). The database contains large cohorts of women who can be followed for seven or eight screening rounds (Fig. 8) and is thus likely to be valuable for evaluating the long-term effects of the screening program. In recent years, the participation rate is slightly lower (Table 3) than Sweden’s average rate of 81%. The large drop in participation between 2009 and 2010 may be at least partly related to the inclusion of younger women (aged 40 or more) beginning in 2010; for example, some women may not have been screened during pregnancy or while breast feeding. As the attendance rate was calculated per year and considered only women invited during that year, younger women (<55 years) with a shorter screening interval (1.5 years) will be overrepresented.

We will continue to collect rich pathology information for more subpopulations, with the goal of having a full coverage in the data set. To facilitate both the development and evaluation of AI cancer detection systems, we aim to add annotations of cancer locations in the images. We have records of radiological appearance for the subset of cancers diagnosed in MBTST, and this may be expanded to the full data set in the future. We also intend to continue expanding the database with retrospective data collection of more recent examinations performed in 2021 and later.

Although the database is important for internal use by offering more convenient access to images and information, it will be even more important for data sharing and collaboration with other researchers. Primarily, we seek active collaboration, in which we can add to the project and build something interesting with other research groups. We may also share anonymous subsets of the data with selected projects, in which we choose not to actively participate. Unfortunately, external researchers cannot directly access the data due to legal, security, and technical concerns. Thus the full database will not be public, but it can be used in collaboration with our research group, e.g., for evaluation of AI systems running at our premises or potentially for federated learning.

The presented database is still in a relatively early stage compared to other mammography databases, such as OMI-DB and CSAW. Because it includes not only screening but also other relevant mammography examinations, however, it offers a more comprehensive resource for evaluating breast cancer screening in the whole target population.

### 4.1 Limitations

The descriptive data presented in the results section is largely preliminary and may change as the database is further curated.

The database has several limitations. It includes examinations from only a single screening center that primarily serves the urban population of Malmö, Sweden, which lessens the generalizability of the data. However, a strength of the database is that Malmö has a multiethnic population, in which one-third is foreign born. At least 183 non-Swedish nationalities are represented, with the largest immigrant groups hailing from Iraq, Syria, Denmark, the former Yugoslavia, and Poland. This can potentially enhance the generalizability of results based on the database, but it is not possible to study specific ethnic groups, as data on ethnicity are not available.

The Malmö breast clinic is a referral center for some surrounding screening centers that include more rural areas; their diagnostic examinations and recalls are thus included but not screening examinations performed elsewhere. These factors together prevent direct estimates of recall rates from the study sample. Data from the biobank SCAN-B, including ABiM, and data from NKBC are currently not linked to the database.

Information on cancer diagnoses was collected from the regional cancer registry, so cancer data are not available for women diagnosed with cancer in another region of Sweden. The plan is to include data from the national cancer registry in the future, but accessing cancer data for women who have moved abroad will be very complicated. The more detailed pathological information in free-text pathology reports is currently available only for selected cases, particularly those diagnosed in 2010 to 2015, but the plan is to gradually expand this number by extracting the same data for all breast cancers in the database. Diagnostic examinations and examinations due to recall from screening are currently not segregated for 2004 to 2008 and parts of 2009. Also screening-detected cancers are not segregated from other cancers in those periods; further data curation is needed to accurately stratify them. The long inclusion period represents both strengths and limitations. Changes in mammography equipment, including shift in manufacturers, over the years can be a strength in developing AI but may also make it harder to compare results over time and may limit the applicability of older data for assessing current practice. Data on specific models of the equipment are currently not available in aggregated form, but it will be collected in near future.

The inclusion of examinations was based on a list of relevant code sequences. Relevant examinations may be missing from the database for two distinct reasons: (1) one or more relevant code sequences were overlooked or (2) the examinations were misfiled under other code sequences. Every effort was made to ensure that the database is complete, and the numbers of various types of examination are reasonable, but it is difficult to guarantee that there are no systematic or unsystematic exclusions. Ultrasound and MRI examinations are not included, which in some cases may be a limitation; in particular, this applies to women with a hereditary high risk of breast cancer who take part in MRI-based screening. As this database is focused on breast cancer screening, it is limited to examinations of women, who are in the target group of breast cancer screening, and thus no mammographic examinations performed on men are included.

Currently, no specific user interface is available for exploring, accessing, and exporting data or for viewing the images, but one may be added in the future. Pixel-level annotation of malignant and benign lesions is planned to be added in the future.

### 4.2 Conclusions

This paper describes how the M-BIG database was designed and implemented as a representative and accessible medical image database containing >450,000 examinations linked to a diverse array of medical data. Future work is needed to add features and curate the existing data.
